# Impact of Frailty on Postoperative Dysphagia in Patients Undergoing Elective Cardiovascular Surgery

**DOI:** 10.1016/j.jacasi.2021.10.011

**Published:** 2022-02-01

**Authors:** Masato Ogawa, Seimi Satomi-Kobayashi, Naofumi Yoshida, Kodai Komaki, Kazuhiro P. Izawa, Mari Hamaguchi, Takeshi Inoue, Yoshitada Sakai, Ken-ichi Hirata, Kenji Okada

**Affiliations:** a Division of Rehabilitation Medicine, Kobe University Hospital, Kobe, Japan; b Department of Public Health, Kobe University Graduate School of Health Sciences, Kobe, Japan; c Division of Cardiovascular Medicine, Department of Internal Medicine, Kobe University Graduate School of Medicine, Kobe, Japan; d Division of Rehabilitation Medicine, Kobe University Graduate School of Medicine, Kobe, Japan; e Division of Cardiovascular Surgery, Department of Surgery, Kobe University Graduate School of Medicine, Kobe, Japan

**Keywords:** cardiac surgery, dysphagia, frailty, postoperative pneumonia, 6MWD, 6-minute walking distance, PED, post-extubation dysphagia, SPPB, Short Physical Performance Battery

## Abstract

**Background:**

Postextubation dysphagia (PED) is a serious postoperative complication following cardiovascular surgery that can lead to a worse prognosis. On the other hand, frailty is a prognostic factor in patients who undergo cardiac surgery.

**Objectives:**

This study investigated the effect of frailty status on PED and impact of PED on postoperative complications.

**Methods:**

This single-center retrospective cohort study included 644 consecutive patients who underwent elective cardiovascular surgery between May 1, 2014, and December 31, 2020; they were assigned to the PED or non-PED group based on postoperative swallowing status, and postoperative complications were investigated. Frailty status and physical functions, including walking speed, grip strength, Short Physical Performance Battery, and 6-minute walking distance, were preoperatively assessed; the frailty-status cutoff for predicting PED was determined from the receiver-operating characteristic curve.

**Results:**

In this study cohort (mean age 67.7 years), the overall PED prevalence was 14.8%; preoperative frailty had a significantly higher prevalence in the PED group (50.0%) than in the non-PED group (20.3%; *P <* 0.001). PED correlated with a higher incidence of postoperative pneumonia and prolonged intensive care unit or hospital stay (*P <* 0.05 for all). After adjustment for confounders, multiple regression analysis revealed that preoperative frailty was independently associated with PED (*P <* 0.001).

**Conclusions:**

PED occurred commonly after cardiovascular surgery and increased the risk of postoperative complications. Preoperative frailty was independently associated with PED. The 6-minute walking distance was the most powerful predictor of PED. Evaluation of preoperative frailty status is important for risk stratification and prevention of postoperative morbidity in patients undergoing surgery.

Dysfunctional deglutition manifests clinically as dysphagia. Dysphagia on extubation following intubation, called postextubation dysphagia (PED), is a serious complication following cardiovascular surgery.^1^ The etiopathogenetic mechanisms underlying PED have not been fully elucidated, although mucosal inflammation, oropharyngeal muscular atrophy, diminished proprioception and sensation, and laryngeal injury associated with prolonged intubation can be attributed to the increase incidence in PED.^2^ A recent systematic review reported that the prevalence of PED after cardiac surgery approached 35% although significant heterogeneity exists.^3^ PED increases mortality, reintubation, the risk of aspiration, and prolongs hospital and intensive care unit stay.^4^ Furthermore, the decreased oral intake associated with PED can worsen the patient’s postoperative nutritional status, leading to further functional decline and worse prognosis.^5^ Thus, the identification of patients who are at risk for PED is critically important to prevent PED.

Frailty has received considerable attention in recent years because of its impact on postoperative mortality, morbidity, and functional decline.^6^ Frailty arises from a multicomponent complex process where the physiological reserve and systemic regulation decline, thereby resulting in a reduced ability to adapt to stressors.^7^ Sarcopenia, which is the loss of muscle mass, is the main cause of frailty.^8^ Interestingly, sarcopenia might cause dysphagia, which is called “sarcopenic dysphagia,” and there is a close relationship between loss of muscle mass and dysphagia.^9^ Thus, frailty, sarcopenia, and/or dysphagia are recognized as geriatric syndromes that have a complex inter-relationship.^10^ However, very few studies have investigated the relationship between preoperative frailty and PED. Thus, this study was conducted to investigate the impact of preoperative frailty on PED in patients who undergo cardiovascular surgery. Furthermore, we also investigated the effect of PED on postoperative complications. Clarifying these relationships and ascertaining the characteristics of physical functions that cause PED will facilitate the development of novel risk-stratification and preoperative intervention options for patients undergoing cardiovascular surgery in a super-aging society.

## Methods

### Study population

**Figure 1:**
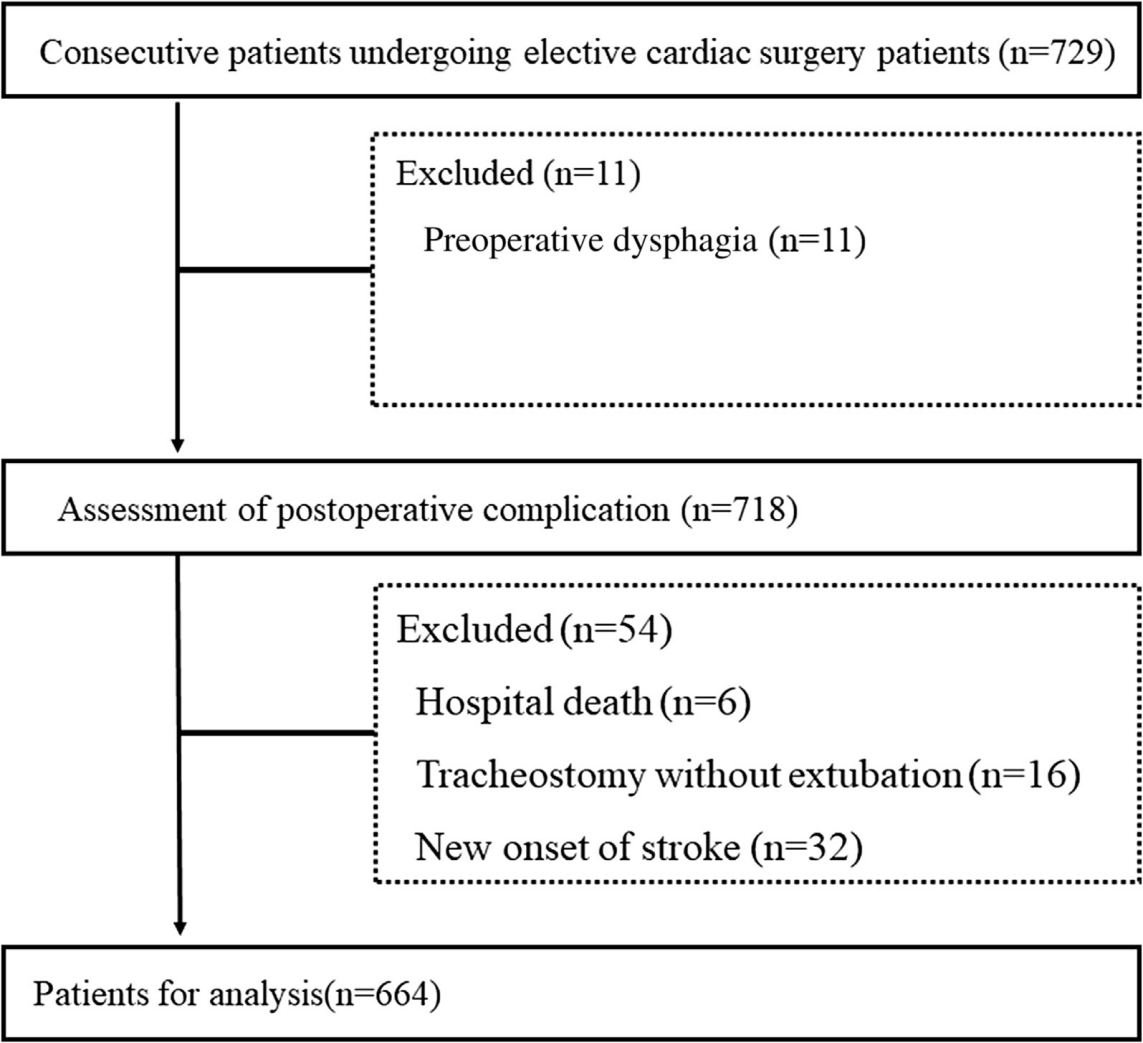
The Patient-Selection Flowchart The flowchart displays the patient flow during the study’s enrollment process.

This retrospective cohort study was conducted from May 1, 2014, to December 31, 2020, at a single university hospital located in an urban area of Japan. We enrolled 729 consecutive inpatients who were able to walk independently before surgery and underwent elective cardiovascular surgery. The exclusion criteria included preoperative dysphagia and postoperative complications, such as hospital death, tracheostomy without postoperative extubation, and new-onset stroke, that make it impossible and/or inappropriate to assess the patient for dysphagia (Figure 1). This study was conducted in compliance with the principles of the Declaration of Helsinki with regard to investigations in human subjects and was approved by the Kobe University Institutional Review Board (approval number B200339). We used the opt-out method for obtaining consent in view of the retrospective study design.

### Clinical characteristics of study participants

We evaluated baseline characteristics, including age; sex; body mass index; comorbidities; left ventricular ejection fraction; New York Heart Association functional class; nutritional status assessed by Mini Nutritional Assessment Short Form^11^; laboratory data, such as the estimated glomerular filtration rate and levels of B-type natriuretic peptide, hemoglobin, and serum albumin; medications; and the EuroSCORE II (European System for Cardiac Operative Risk Evaluation II).^12^ Laboratory data were evaluated within 1 week before the surgery. We recorded operative variables, including the type and duration of surgery and ventilation, hospital mortality, postoperative surgery-related complications, length of intensive care unit stay, length of hospital stay, and discharge locations. In our center, all patients scheduled for surgical intervention underwent oral assessment. The presence of loose teeth at high risk of avulsion during intubation for surgery was carefully evaluated. After cardiovascular surgery and during the postoperative period, patients received routine professional oral management by dental hygienists and daily mouth cleaning by nurses.

### Assessment of dysphagia

The swallowing status was assessed by the Food Intake Level Scale.^13^ Based on the Food Intake Level Scale score, dysphagia was categorized as no oral intake (score: 1-3), oral intake and alternative nutrition (score: 4-6), and oral intake alone (score: 7-10). The Food Intake Level Scale assessment was undertaken 3 times as follows: 1) within 1 week before surgery; 2) when postoperative oral intake was permitted after the attending cardiologists had confirmed that the patient’s circulatory status was stable; and 3) immediately before hospital discharge. Following postoperative extubation, a bedside swallowing evaluation of the level of consciousness, mouth or tongue function, vocal function, oral hygiene, and cough reflex was performed by well-trained nurses who specialized intensive care unit nursing. Then, a water-swallowing test was performed for patients who were considered to have sufficient swallowing ability. Based on the results of these tests, the eating pattern was determined. In this study, we defined dysphagia as a Food Intake Level Scale score ≤7, as described previously.^14^ We excluded patients with a preoperative Food Intake Level Scale score ≤7 from the analysis. Furthermore, we investigated the timing of the postoperative Food Intake Level Scale assessment and the time point when the first oral solid-food intake was initiated. We evaluated the RODICS (Risk of Dysphagia in Cardiac Surgery) score, which is used to identify patients at high risk for postoperative dysphagia.^15^

### Assessment of frailty and physical function

We assessed the preoperative frailty status within the week before the intervention using the Japanese version of the Cardiovascular Health Study frailty index.^16^ The frailty phenotype is based on the following 5 components: slowness (gait speed: <1.0 m/s), weakness (grip strength: <28 kg for men and <18 kg for women), weight loss (>2 kg in the past 6 months), exhaustion, and low physical activity; 3 of the 5 criteria are required for a diagnosis of frailty. Physical performance was assessed using Short Physical Performance Battery (SPPB).^17^ SPPB is a strongly recommended test with respect to reliability, validity, feasibility, and predictive value after cardiac surgery.^17^^,^^18^ The SPPB includes usual walking speed over 4 m, 5 chair-stands test, and balance test. Gait speed was measured by positioning the patient at the starting point of a 4-m course, instructing them to walk at their normal pace, and using a stopwatch to record the time required to complete the course. The psoas muscle index was used as an index of total skeletal muscle mass. To calculate the psoas muscle index, the outer margin of the major psoas muscle cross section at the level of the caudal end of the L3 vertebral body was manually traced on abdominal computed tomography images, and the sum of the left and right cross-sectional areas was divided by the height squared.^19^ For evaluating exercise capacity, the 6-minute walking distance (6MWD) was assessed by a trained physiotherapist who is a registered instructor of cardiac rehabilitation.^20^

### Statistical analysis

The sample size to detect 15% difference in prevalence of PED between the 2 groups was determined by using the formula with α = 0.05, statistical power = 0.80, effect size = 0.20, and the required sample size was 566 patients. After confirming the normal distribution of data using the Shapiro-Wilk test, we conducted statistical analyses. Participants were assigned to the PED or non-PED groups, and intergroup differences in the baseline clinical characteristics were determined using the independent Student’s *t*-test and chi-square test. The results are reported as mean ± SD for parametric data and as the median (interquartile range) for nonparametric data. To analyze factors that affect dysphagia, a logistic regression analysis was used to examine each association between the incidence of dysphagia and each variable with the incidence of dysphagia as the dependent variable, whereas the clinical characteristics were independent variables. Receiver-operating characteristic curves were constructed by plotting true-positive rates (sensitivity) against false-positive rates (1 − specificity) to determine the best cutoff value for each variable. The area under the curve was calculated from the receiver-operating characteristic curve for each variable, and the cutoff value was calculated based on the Youden index.^21^ Age, sex, and other factors in the univariate analysis with a *P* value <0.10 were entered simultaneously in a multivariate logistic regression model. To confirm the results, we reduced the risk of bias by adjusting for baseline characteristics using propensity scores, which were obtained via a logistic regression analysis with dysphagia as the dependent variable and the 26 variables listed in Table 1, except frailty, and perioperative characteristics, such as ventilation time, operative time, and procedure type, as independent variables (C-statistic = 0.721). We performed a 1:1 nearest available neighbor matching on the logit of the propensity score with a caliper value of 0.2 and no replacement. All statistical analyses were performed using R (The R Foundation for Statistical Computing). The statistical significance level was set at *P <* 0.05.

**Table 1:** Baseline Clinical Characteristics of the Patients With or Without PED

	Total (N = 664)	PED Group (n = 98)	Non-PED Group (n = 566)	SMD	*P* Value
Age, y	67.72 ± 13.69	73.62 ± 9.78	66.55 ± 14.12	0.56	<0.001
Female	282 (42.5)	46 (46.9)	236 (41.7)	0.38	0.376
BMI, kg/m^2^	22.86 ± 3.79	22.30 ± 3.71	22.97 ± 3.80	0.18	0.111
Lab data					
Albumin, g/dL	3.82 ± 0.87	3.51 ± 1.01	3.87 ± 0.84	0.39	<0.001
BNP, pg/mL	105.0 (45.0-228.0)	148.5 (56.5-283.8)	100.0 (44.0-220.5)	0.23	0.059
Hemoglobin, g/dL	12.49 ± 2.50	11.39 ± 2.82	12.69 ± 2.39	0.50	<0.001
eGFR, mL/min/1.73 m^2^	56.11 ± 24.40	44.69 ± 24.78	58.09 ± 23.81	0.55	<0.001
Comorbidity					
Diabetes	129 (19.4)	12 (12.2)	117 (20.7)	0.23	0.053
COPD	96 (14.4)	13 (13.3)	83 (14.7)	0.04	0.876
Hypertension	407 (61.3)	66 (67.3)	341 (60.2)	0.15	0.121
Hemodialysis	19 (2.9)	7 (7.1)	12 (2.1)	0.24	0.006
Dyslipidemia	255 (38.4)	40 (40.8)	215 (38.0)	0.06	0.101
Previous cardiac surgery	91 (13.7)	21 (21.4)	70 (12.4)	0.25	0.037
LVEF, %	58.55 ± 14.44	59.64 ± 12.30	60.23 ± 10.96	0.11	0.661
NYHA functional class				1.05	<0.0001
I	199 (30.0)	172 (30.4)	27 (27.6)		
II	382 (57.5)	333 (58.8)	49 (50.0)		
III	83 (12.5)	61 (10.8)	22 (22.4)		
MNA-SF				0.532	<0.001
Good	462 (69.6)	48 (49.0)	414 (73.1)		
At risk	145 (21.8)	32 (32.7)	113 (20.0)		
Malnutrition	57 (8.6)	18 (18.3)	39 (6.9)		
EuroSCORE II	4.52 ± 3.75	6.63 ± 4.95	4.15 ± 3.37	0.58	<0.001
Medications					
β-blocker	414 (64.3)	53 (54.0)	361 (63.8)	0.29	0.111
ACE inhibitor	100 (15.1)	11 (11.2)	89 (15.7)	0.15	0.399
ARB	235 (35.4)	41 (41.8)	194 (34.3)	0.15	0.157
Statin	200 (30.1)	27 (27.6)	173 (30.6)	0.08	0.356
Diuretic	245 (36.9)	38 (38.8)	207 (36.6)	0.04	0.374
Frailty	164 (24.7)	49 (50.0)	115 (20.3)	0.65	<0.001
Gait speed, m/s	1.05 ± 0.27	0.95 ± 0.28	1.08 ± 0.26	0.42	0.0001
Grip strength, kg	26.52 ± 9.44	22.57 ± 8.00	27.14 ± 9.50	0.52	<0.001
SPPB, point	10.89 ± 2.10	9.85 ± 3.01	11.06 ± 1.86	0.48	<0.001
6MWD, m	399.09 ± 115.09	328.59 ± 106.76	411.18 ± 112.20	0.75	<0.001
PMI, cm^2^/m^2^	5.35 ± 1.72	4.27 ± 1.28	5.59 ± 1.68	0.43	<0.001
RODICS score	11.94 ± 4.73	14.25 ± 5.73	11.54 ± 4.42	0.53	<0.001

Values are mean ± SD, n (%), or median (interquartile range).

6MWD = 6-minute walking distance; ACE = angiotensin-converting enzyme; ARB = angiotensin II receptor blocker; BMI = body mass index; BNP = B-type natriuretic peptide; COPD = chronic obstructive pulmonary disease; eGFR = estimated glomerular filtration rate; EuroSCORE II = European System for Cardiac Operative Risk Evaluation II; LVEF = left ventricular ejection fraction; MNA-SF = Mini Nutritional Assessment Short Form; NYHA = New York Heart Association; PED = postextubation dysphagia; PMI = psoas muscle index; RODICS = Risk of Dysphagia in Cardiac Surgery; SMD = standardized mean differences; SPPB = Short Physical Performance Battery.

## Results

### Baseline characteristics

Based on the study enrollment criteria, a total of 664 individuals (43% female) with mean age 67.7 ± 13.7 years were included in the present study (Figure 1). In our cohort, PED developed in 98 patients (14.8%). Baseline characteristics according to the dysphagia grouping are shown in Table 1. Patients in the PED group were significantly older than those in the non-PED group (*P <* 0.001). Patients with dysphagia had lower serum albumin, hemoglobin, and estimated glomerular filtration rate levels than patients who were nondysphagic did (*P <* 0.05 for all). Furthermore, nutritional status, as indicated by the Mini Nutritional Assessment Short Form score, was significantly poorer in the PED group than in the non-PED group (*P <* 0.001). With regard to comorbidities, the proportion of patients who had undergone previous cardiac surgery was much higher in the PED group than in the non-PED group (*P <* 0.05 for all). The change in the Food Intake Level Scale score from before to after the surgery is shown in the Central Illustration and Supplemental Figure S1. The mean initial postoperative Food Intake Level Scale score was 8.11 ± 1.55, but there was significant improvement at discharge (8.51 ± 1.05). However, the Food Intake Level Scale score was significant lower in the PED group compared with in the non-PED group even at discharge (Table 2). The prevalence of frailty was 24.7% in the study cohort and was significantly higher in the PED group than in the non-PED group (50.0% vs 20.3%; *P <* 0.001). Furthermore, the intergroup difference in the prevalence of frailty between patients with and without dysphagia did not change after propensity score matching (Central Illustration, Supplemental Table S1). Each physical function parameter, such as gait speed, grip strength, SPPB, and 6MWD, was significantly decreased in the PED group compared with that in the non-PED group (*P <* 0.05 for all).

**Central Illustration:**
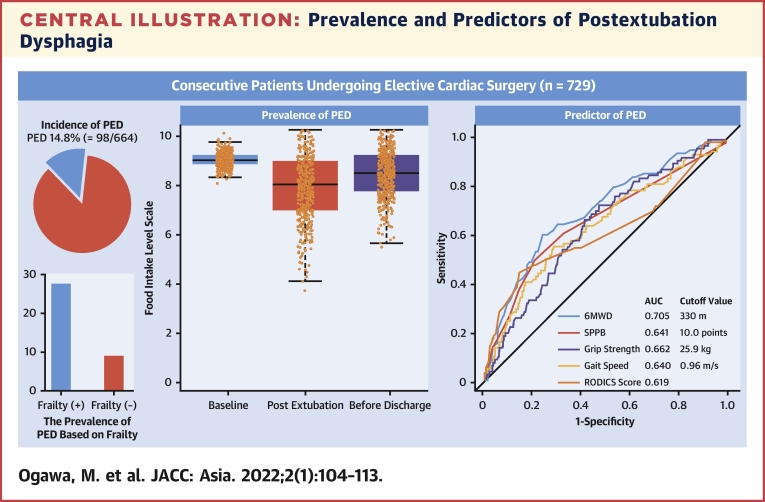
Prevalence and Predictors of Postextubation Dysphagia **(Left)** Incidence of postextubation dysphagia (PED) was 14.8%, which was significantly higher in the frail group than that in the nonfrail group. **(Middle)** Longitudinal changes in the preoperative and postoperative PED are shown. Before discharge, 5.8% of patients still had PED. **(Right)** In receiver-operating characteristic curves to predict the incidence of PED, 6-minute walking distance (6MWD) was the most predictable parameter among physical functions. AUC = area under the curve; RODICS = Risk of Dysphagia in Cardiac Surgery; SPPB = Short Physical Performance Battery.

**Table 2:** Comparison of the Postoperative Course of Participants With or Without PED

	Total (N = 664)	PED Group (n = 98)	Non-PED Group (n = 566)	SMD	*P* Value
Ventilation time, h	13.60 ± 20.50	31.35 ± 37.70	10.66 ± 13.96	0.73	<0.001
Operative time, h	364.53 ± 123.50	409.96 ± 154.25	356.69 ± 115.75	0.39	<0.001
Procedure type				0.47	<0.001
Aortic	88 (13.3)	27 (27.6)	61 (10.8)		
CABG	88 (13.3)	9 (9.2)	79 (14.0)		
Valve	434 (65.4)	52 (53.1)	382 (67.5)		
Combination	54 (8.1)	10 (10.2)	44 (7.8)		
Pneumonia	20 (3.0)	16 (16.3)	4 (0.7)	0.67	<0.001
Infection-related complications	22 (3.3)	4 (4.1)	18 (3.2)	0.55	0.05
Renal failure	25 (3.7)	12 (12.2)	13 (2.3)	0.40	<0.001
Delirium	211 (31.8)	80 (81.6)	131 (23.1)	0.85	<0.001
Postoperative AF	291 (43.8)	45 (45.9)	246 (43.5)	0.06	0.65
ICU stay, d	3.23 ± 2.41	6.62 ± 6.30	2.79 ± 1.46	0.96	<0.001
Hospital stay, d	21.44 ± 10.42	30.01 ± 12.64	20.02 ± 9.28	0.90	<0.001
Discharge location, home	564 (84.9)	44 (44.9)	520 (91.9)	1.18	<0.001
Preoperative FILS score	9.11 ± 0.30	9.34 ± 0.29	9.48 ± 0.20	0.10	0.66
Postoperative FILS screening time, d	1.66 ± 1.10	2.65 ± 1.51	1.50 ± 0.93	0.94	<0.001
Time to the first meal, d	1.88 ± 1.13	2.95 ± 1.52	1.70 ± 0.95	0.99	<0.001
FILS score at discharge	8.51 ± 1.05	7.23 ± 1.45	9.31 ± 0.57	1.88	<0.001

Values are mean ± SD or n (%).

AF = atrial fibrillation; CABG = coronary artery bypass graft; FILS = Food Intake LEVEL Scale; ICU = intensive care unit; other abbreviations as in Table 1.

### Operative profiles

Operative profiles, postoperative complications, and the clinical course, in patients with or without PED, are shown in Table 2. The duration of surgery and postoperative ventilation was significantly longer in the PED group compared with in the non-PED group (*P <* 0.001). In the comparison of surgical procedures, the PED group had a higher rate of aortic surgery, whereas the non-PED group had a higher rate of coronary artery bypass graft.

### Postoperative outcomes

Postoperative pneumonia and delirium developed more often in the PED group than in the non-PED group (*P <* 0.001 for all). More patients in the PED group needed longer intensive care unit and hospital stays than patients in the non-PED group did (*P <* 0.001 for all). The first postoperative evaluation of swallowing was undertaken 1.7 ± 1.1 days after surgery, and oral intake was initiated 1.9 ± 1.1 days after surgery; both durations were prolonged in the PED group (*P <* 0.05 for all).

### Predictors of PED

Table 3 shows the results of the evaluation of risk factors for PED. In the multiple logistic regression analysis, aging, aortic surgery, frailty, and duration of postoperative ventilation time were independently associated with PED. The receiver-operating characteristic curve for predicting PED is shown in Supplemental Figure S2. For each parameter, the 6MWD had the highest predictive power for PED (AUC: 0.71) (Central Illustration, Supplemental Figure S2). The cutoff values for each physical function were as follows: gait speed: 0.96 m/s; SPPB: 10.0 points; grip strength: 25.9 kg; 6MWD: 330.0 m.

**Table 3:** Univariate and Multivariable Analysis of the Predictive Factors for the Development of PED

	Univariate Model		Multivariable Model	
	OR (95% CI)	*P* Value	OR (95% CI)	*P* Value
Age	1.05 (1.03-1.08)	<0.0001	1.05 (1.02-1.09)	0.001
Female	1.24 (0.80-1.90)	0.33	1.09 (0.59-2.03)	0.76
Previous cardiac surgery, yes	1.96 (1.12-3.34)	0.0195	1.72 (0.73-3.89)	0.21
eGFR	0.98 (0.97-0.99)	<0.0001	1.00 (0.99-1.02)	0.90
Hemoglobin	0.84 (0.78-0.91)	<0.0001	0.92 (0.80-1.08)	0.30
MNA-SF, at risk and/or malnutrition	3.08 (1.62-5.66)	0.0004	1.39 (0.55-3.40)	0.47
NYHA functional class	1.44 (1.01-2.06)	0.04	0.94(0.54-1.62)	0.82
EuroSCORE II	1.14 (1.09-1.21)	<0.0001	1.06 (0.98-1.15)	0.16
Type, aortic	3.14 (1.86-5.24)	<0.0001	5.05 (2.12-12.14)	0.003
Frailty, yes	3.90 (2.40-6.35)	<0.0001	2.79 (1.46-5.36)	0.002
Ventilation time	1.04 (1.03-1.05)	<0.0001	1.04 (1.03-1.06)	<0.0001

OR = odds ratio; other abbreviations as in Table 1.

## Discussion

The main findings of this study were that 14.8% of the participants developed PED after cardiac surgery, and preoperative frailty was found to be an independent predictor of PED. This is the first study to demonstrate that physical functions, such as gait speed, grip strength, SPPB, and 6MWD, significantly influenced the incidence of PED; the 6MWD, compared with the established swallowing risk score, was the most powerful predictive indicator of PED.

In this study, the prevalence of PED was 14.8%, which was similar to the prevalence reported in previous studies.^15^^,^^22^ The method of dysphagia assessment differed among previous studies, and the incidence of PED varies by the method of assessment.^1^^,^^23^ Videofluoroscopic swallow study or a fiberoptic endoscopic evaluation of swallowing are the gold standard for diagnosing swallowing disability, although these assessments are unsuitable for routine use in the clinical setting because of their invasiveness and complexity.^23^ Thus, the bedside swallowing evaluation is the primary assessment tool for determining the swallowing function in the clinical setting despite its limitations with regard to its ability to accurately assess the severity of aspiration and to guide prognosis.^23^ Therefore, we focused on indicators of the functional impact of dysphagia-related symptoms with regard to the oral intake of food or liquid, such as the Food Intake Level Scale score. It is noteworthy that high prevalence of PED was apparent, although silent aspiration may not have been identified.

Most patients experienced spontaneous recovery of swallowing function during their hospital stay. Interestingly, when dysphagia developed, even temporarily, postoperative complications, such as pneumonia and reintubation, developed in patients with PED. Recently, Plowman et al^24^ demonstrated that the patients with PED had prolonged delay in the resumption of oral intake, incurred additional hospital costs, and had a longer hospital stay. Furthermore, patients with PED had a prolonged duration to the initiation of oral intake, which could result in functional decline and worse prognosis.^5^ These findings support the inference that even transient PED can result in fatal complications and long-term physical and psychological damage. The results of the present study suggest the importance of the postoperative evaluation of deglutition and interventions undertaken by deglutition-rehabilitation specialists, such as the speech language pathologist, in a timely manner.

The relationship between cerebrovascular disease and dysphagia is well documented, and a causative role is attributed to dysphagia.^25^ Therefore, we excluded patients with postoperative new-onset stroke. Nevertheless, after excluding patients who had had a stroke, preoperative frailty was independently associated with PED following cardiovascular surgery. Furthermore, frailty was a more powerful predictor of PED than the RODICS score was (Central Illustration). One possible explanation is that vulnerability appears and becomes increasingly problematic after highly invasive surgery, despite the absence of preoperative symptoms of frailty.^6^ In particular, dysfunction of deglutition could be one of the commonest problems that is postoperatively exposed. In other words, the clinical dysphagia of patients who are frail was unmasked by the surgery.^26^ In patients who underwent cardiac surgery, the functional capacity declined by approximately 20% in the perioperative period because of postoperative systemic inflammation and protein catabolism.^27^ A perioperative rapid decline in generalized skeletal muscle strength can have repercussions leading to weakness of the deglutition muscles, including the lingual and mylohyoid muscles, in patients who are frail.^28^ It is well known that loss of teeth and poor oral hygiene contribute to the development of deglutition dysfunction, thereby decreasing the efficiency of chewing and further worsening the swallowing dysfunction.^28^ We previously reported that poor oral status was closely related to physical frailty.^29^ The multidimensional components of frailty may manifest as dysphagia. Dysphagia can lead to malnutrition, which in turn can lead to weight loss, decreased healing ability, and increased incidence of other diseases.^30^ The definitive evidence presented here suggests that preoperative interventions for frailty may prevent PED, thus breaking the vicious cycle of frailty and dysphagia.

In addition, aortic surgery or prolonged ventilation were independent predictors of PED in this study. The left recurrent nerve, which runs under the aortic arch, was frequently injured during aortic surgery, which resulted in unilateral vocal cord immobility.^31^ Approximately 60% of the patients with unilateral vocal cord immobility developed difficulty in swallowing. Furthermore, prolonged ventilation causes direct trauma by the endotracheal and tracheostomy tubes and diminishes the laryngeal sensory function.^32^ These mechanisms possibly contributed to the development of PED. However, this study did not investigate vocal cord immobility in detail and, thus, further research into this aspect is needed.

Previous studies have investigated the relationship between frailty or sarcopenia and dysphagia in older patients who are hospitalized and in community-dwelling older individuals.^33^^,^^34^ Deglutition difficulties caused by sarcopenia are diagnosed as sarcopenic dysphagia; however, this condition is difficult to diagnose because of the very slow progression and atypical presentations of dysphagia.^30^ Thus, dysphagia caused by an age-related decrease in muscle mass tends to be underestimated and underdiagnosed.^30^ Our results for each of the preoperative physical functions and cutoff values that predicted PED facilitate the identification of patients who are at risk for dysphagia, which is difficult to diagnose preoperatively. Of note, frailty is a reversible clinical condition if treated early. Steinmetz et al^35^ concluded, based on the findings of their randomized controlled trial, that exercise-based prehabilitation is effective in improving functional capacity and quality of life in patients scheduled to undergo elective cardiac surgery. Recent studies investigated the beneficial effects of preoperative nutritional intervention in patients who underwent hepatic resection or had cancer surgery, whereas no reports have described the effects of preoperative nutritional intervention for patients undergoing cardiovascular surgery.^36^, ^37^, ^38^ Enhanced recovery protocols, such as prehabilitation and nutritional intervention could improve the frailty status and help prevent PED.

### Study limitations

First, this study was a nonrandomized, retrospective observational study based on single-center design and small sample size, which may affect the external validity of the results. Second, we focused on the functional impact of dysphagia-related symptoms by using the Food Intake Level Scale score and only used the water-swallowing test to screen for dysphagia. Thus, patients with silent aspiration may not have been identified because the test only evaluated the pharyngeal swallow function without mastication. Third, the follow-up period was the duration of the hospital stay. Therefore, the effect of PED on long-term prognosis could not be determined. Fourth, we cannot demonstrate the relationship between sarcopenia and dysphagia, though we found a significant relationship between muscle mass and dysphagia. Future longitudinal studies on the assessment of sarcopenia and dysphagia were warranted. Despite these limitations, this is the largest study to demonstrate the effects of preoperative frailty on PED.

## Conclusions

Preoperative frailty was independently associated with PED following cardiovascular surgery. PED occurred frequently and was associated with postoperative complications, even if the condition was transient. 6MWD was the most powerful predictive indicator of PED. The clinical effects of PED on long-term outcomes or functional status should be investigated in a future well-designed large-scale clinical study.

Perspectives **COMPETENCY IN MEDICAL KNOWLEDGE:** This study adds to medical knowledge and patient care in that it demonstrates that postextubation dysphagia was associated with postoperative complications, even if the patient is undergoing cardiac surgery. Preoperative frailty was independently associated with postextubation dysphagia following cardiovascular surgery.

**TRANSLATIONAL OUTLOOK:** Frailty assessment has the potential to improve the risk stratification for postextubation dysphagia in the patients with cardiac surgery.

## Funding Support and Author Disclosures

This work was supported by the Japan Society for the Promotion of Science KAKENHI (grant JP20K19447). This funding source had no role in the design of this study and will not have any role during its execution, analyses, interpretation of the data, or decision to submit results. The data that support the findings of this study are available from the corresponding author, Dr. Satomi-Kobayashi upon reasonable request. The authors have reported that they have no relationships relevant to the contents of this paper to disclose.
