# Supplementary material for: Impact of Frailty on Postoperative Dysphagia in Patients Undergoing Elective Cardiovascular Surgery
Source: JACC Asia. 2022 Feb 1;2(1):104–13. doi: 10.1016/j.jacasi.2021.10.011 (PMC9627818; doi:10.1016/j.jacasi.2021.10.011)
Supplement: Supplemental Figures 1 and 2 and Supplemental Table 1 [file mmc1.rtf]

Impact of Frailty on Postoperative Dysphagia in Patients Undergoing Elective Cardiovascular Surgery
Running title: Postoperative Dysphagia in Frail Cardiovascular Surgery Patients
Masato Ogawa, PT, PhD,a,b Seimi Satomi-Kobayashi, MD, PhD,c Naofumi Yoshida, MD, PhD,c Kodai Komaki, PT,a Kazuhiro P. Izawa, PT, PhD,b Mari Hamaguchi, MD,e Takeshi Inoue, MD,e Yoshitada Sakai, MD, PhD,d Ken-ichi Hirata, MD, PhD,c Kenji Okada, MD, PhDe
Supporting information


Supplemental Table S1. Comparison of Clinical Outcomes With or Without Postoperative Dysphagia in a Propensity-matched Study Population

Variables	PED Group
(n = 58)	Non-PED Group (n = 58)	SMD	P Value	
Frailty, n (%)	29 (50.0)	16 (27.6)	0.652	0.013	
Pneumonia, n (%)	10 (17.2)	0 (0.0)	0.667	0.009	
Infection-related complications, n (%)	3 (5.2)	1 (1.7)	0.048	0.31	
ICU stay, days	5.40 ± 4.33	3.20 ± 1.48	0.957	0.0005	
Hospital stay, days	29.6 ± 12.0	21.9 ± 10.4	0.902	0.0006	
Postoperative AF, n (%)	29 (44.6)	20 (36.4)	0.055	0.08	
Discharge location, home	28 (48.3)	46 (79.3)	1.184	0.0005	
AF, atrial fibrillation; ICU, intensive care unit; PED, postoperative dysphagia; SMD, standardized mean differences. 


Supplemental Figure S1. Longitudinal Changes in the Preoperative and Postoperative FILS
PED was observed 14.5%, which defined PED as an FILS ≤7. Before discharge, 5.8% remained PED. FILS, Food Intake Level Scale; PED, post-extubation dysphagia.


Supplemental Figure S2. ROC Curves to Predict the Incidence of PED
ROC curve for the physical function and RODICS score to predict the incidence of PED. Values in the figures are AUC. 6MWD was the most predictable parameter among physical function. 6MWD, 6-minute walking distance; AUC, area under the curve; PED, postextubation dysphagia; ROC, receiver-operating characteristic; RODICS, Risk of Dysphagia in Cardiac Surgery. 
